# The Interplay Between Tumor Suppressor p53 and Hypoxia Signaling Pathways in Cancer

**DOI:** 10.3389/fcell.2021.648808

**Published:** 2021-02-18

**Authors:** Cen Zhang, Juan Liu, Jianming Wang, Tianliang Zhang, Dandan Xu, Wenwei Hu, Zhaohui Feng

**Affiliations:** Department of Radiation Oncology, Rutgers Cancer Institute of New Jersey, Rutgers-State University of New Jersey, New Brunswick, NJ, United States

**Keywords:** hypoxia, HIF, tumor suppressor, p53, mutant p53, cancer, cancer therapy

## Abstract

Hypoxia is a hallmark of solid tumors and plays a critical role in different steps of tumor progression, including proliferation, survival, angiogenesis, metastasis, metabolic reprogramming, and stemness of cancer cells. Activation of the hypoxia-inducible factor (HIF) signaling plays a critical role in regulating hypoxic responses in tumors. As a key tumor suppressor and transcription factor, p53 responds to a wide variety of stress signals, including hypoxia, and selectively transcribes its target genes to regulate various cellular responses to exert its function in tumor suppression. Studies have demonstrated a close but complex interplay between hypoxia and p53 signaling pathways. The p53 levels and activities can be regulated by the hypoxia and HIF signaling differently depending on the cell/tissue type and the severity and duration of hypoxia. On the other hand, p53 regulates the hypoxia and HIF signaling at multiple levels. Many tumor-associated mutant p53 proteins display gain-of-function (GOF) oncogenic activities to promote cancer progression. Emerging evidence has also shown that GOF mutant p53 can promote cancer progression through its interplay with the hypoxia and HIF signaling pathway. In this review, we summarize our current understanding of the interplay between the hypoxia and p53 signaling pathways, its impact upon cancer progression, and its potential application in cancer therapy.

## Introduction

Extensive studies have established that p53 is a key tumor suppressor, and disruption of the p53 function is a prerequisite for the initiation and/or progression of many human cancers. The key role of p53 in tumor suppression has been clearly demonstrated by the following evidence. The p53 gene is mutated in over half of all human cancers and almost in every type of human cancer (Vousden and Prives, 2009; Muller and Vousden, 2014; Donehower et al., 2019; Levine, 2019; Zhang et al., 2020). Li–Fraumeni syndrome, a rare autosomal-dominant disorder caused by germline p53 mutations, predisposes patients to different types of tumors, including breast cancer, soft-tissue and bone sarcomas, and brain tumors (Malkin et al., 1990; Srivastava et al., 1990). p53 knockout mice are extremely prone to develop tumors, mainly lymphomas and sarcomas (Donehower et al., 1992; Attardi and Jacks, 1999). In addition to DNA mutations, different mechanisms have been reported to disrupt the tumor suppressive function of p53 in cancer and lead to tumorigenesis, including overexpression of critical p53 negative regulators, such as MDM2, MDM4, and WIP1, and inactivation of p53 by oncoproteins encoded by different tumor viruses, such as simian virus 40 large T antigen, and E6 of human papilloma virus (Vousden and Prives, 2009; Muller and Vousden, 2014; Donehower et al., 2019; Levine, 2019; Zhang et al., 2020).

Tissue hypoxia results from the inadequate supply of oxygen (O_2_) that compromises the biological functions of cells. Most solid tumors have regions under permanent or transient hypoxic conditions resulting from the poor blood supply with aberrant vascularization (Schito and Semenza, 2016; Petrova et al., 2018; Choueiri and Kaelin, 2020; de Heer et al., 2020; Lee et al., 2020). Hypoxia activates the hypoxia signaling pathway, which is predominantly governed by hypoxia-inducible factor (HIF), leading to a series of cellular responses, including cell death, survival, angiogenesis, metabolic reprogramming, metastasis, stemness, inflammation, immune evasion, etc., to help tumor cells to adapt to the hypoxic environment and contribute to tumor progression (Schito and Semenza, 2016; Petrova et al., 2018; Choueiri and Kaelin, 2020; de Heer et al., 2020; Lee et al., 2020). Loss of the tumor-suppressive function of p53 and hypoxia are two common biological events in solid tumors, and therefore, p53 and hypoxia in cancer have been extensively studied. Many studies have demonstrated the close but complex interplay between the p53 and the hypoxia signaling pathways and its impact upon tumor progression, which are summarized in this review.

## The p53 Signaling Pathway

p53 is a transcription factor, and mainly functions through transcriptional regulation of its target genes, although p53 can also directly interact with some other proteins to regulate cellular processes (e.g., apoptosis and metabolism). Usually, p53 protein is maintained at low levels with a very short protein half-life under non-stressed conditions in normal cells and tissues. p53 can respond to a wide variety of stress signals, including DNA damage, nutritional deprivation, impaired ribosome biogenesis, activation of oncogenes, as well as hypoxia. In response to these stress signals, p53 protein half-life is dramatically increased, leading to p53 protein accumulation and activation in cells. Once p53 is activated, p53 binds to the p53-binding elements in its target genes to selectively transcribe these genes. A myriad of protein-coding genes and many non-coding genes, including microRNAs (miRNAs) and long non-coding RNAs (LncRNAs), have been identified as p53 target genes (Feng and Levine, 2010; Liu et al., 2017a; Dangelmaier et al., 2019; Levine, 2019). Through selective transcriptional induction or repression of these target genes, p53 regulates various cellular responses, including cell cycle arrest, senescence, apoptosis, autophagy, ferroptosis, DNA repair, metabolism, cell migration/invasion, modulation of oxidative stress, etc., which contribute to the role of p53 in tumor suppression (Vousden and Prives, 2009; Muller and Vousden, 2014; Levine, 2019; Liu J. et al., 2019; Liu et al., 2020; Zhang et al., 2020). Besides of the role of p53 in tumor suppression, p53 has also been shown to play important roles in many other biological and pathological processes, such as anti-infection, immune response, maternal reproduction, development, metabolic diseases, ischemia and tissue injuries, neurodegeneration, and aging (Hu, 2009; Levine and Oren, 2009; Vousden and Prives, 2009; Muller and Vousden, 2014; Levine, 2019; Liu J. et al., 2019; Zhang et al., 2020).

p53 protein levels and activities are under tight regulation in cells to ensure that p53 can function properly in these fundamental cellular processes. The post-translational protein modifications, including the ubiquitination modification, play the most critical role in the regulation of the levels and activities of p53 protein (Hock and Vousden, 2014; Meek, 2015; Levine, 2019; Liu Y. et al., 2019). The E3 ubiquitin ligase MDM2 is the most critical negative regulator of p53, which directly binds to p53 and ubiquitinates p53 for proteasomal degradation to maintain p53 protein at a low level under the non-stressed condition (Zhao et al., 2014; Karni-Schmidt et al., 2016; Haupt et al., 2019). In addition to mediating proteasomal degradation of p53, MDM2 also promotes p53 nuclear export to reduce the binding of p53 to its target genes in the nucleus, leading to the downregulation of the p53 transcriptional activities. Interestingly, MDM2 is a p53 target gene that can be upregulated by p53. In response to stress signals, p53 is disassociated from MDM2, leading to p53 protein accumulation and activation in cells. Once p53 is activated, MDM2 expression is induced by p53, which in turn downregulates p53 levels and activities. Thus, MDM2 forms a negative feedback loop with p53 to keep p53 levels under tight control in cells (Zhao et al., 2014; Karni-Schmidt et al., 2016; Haupt et al., 2019). MDM4 (also known as MDMX), a structural homolog of MDM2 that lacks the E3 ubiquitin ligase activity, is an additional important negative regulator of p53. MDM4 negatively regulates p53 in both MDM2-dependent and -independent manners; MDM4 interacts with MDM2 and promotes MDM2−mediated ubiquitination and degradation of p53, and also interacts with p53 to suppress the transcriptional activities of p53 (Zhao et al., 2014; Karni-Schmidt et al., 2016; Haupt et al., 2019). Both MDM2 and MDM4 are overexpressed in a variety of tumors, which result in the downregulation of p53 protein levels and activities, leading to tumor initiation and/or progression (Karni-Schmidt et al., 2016; Donehower et al., 2019; Haupt et al., 2019). Furthermore, p53 has also been reported to be ubiquitinated for proteasomal degradation by many other E3 ubiquitin ligases, such as Pirh2, COP1, CHIP, TRIM32, etc. (Hock and Vousden, 2014; Levine, 2019; Liu Y. et al., 2019). In addition to ubiquitination, other post-translational modifications, including phosphorylation, acetylation, methylation, neddylation, and SUMOylation, have also been reported to play important roles in the regulation of protein levels and transcriptional activities of p53 (Hock and Vousden, 2014; Levine, 2019; Liu Y. et al., 2019). Many proteins are involved in these post-translational modifications of p53. Further, different positive and negative feedback loops are formed between p53 and p53 regulators to tightly regulate p53 levels and activities (Hock and Vousden, 2014; Levine, 2019; Liu Y. et al., 2019).

## Hypoxia and Hif Signaling Pathway

Hypoxia refers to a condition in which oxygen levels are limited in a tissue, and is associated with both physiological and pathological conditions in humans. Hypoxia can result from insufficient blood flow to specific organs, low levels of hemoglobin, or exposure to chemical compounds. Hypoxia in tumors is caused by a variety of mechanisms (Schito and Semenza, 2016; Petrova et al., 2018; Choueiri and Kaelin, 2020; de Heer et al., 2020; Lee et al., 2020). For instance, tumor perfusion hypoxia arises from an abnormal disorganization of tumor vasculature, characterized by structural, functional, and cellular abnormalities and inadequate blood flow, leading to transient ischemia. Tumor diffusion hypoxia is caused by long oxygen diffusion distances between tumor cells and blood vessels, and blood flow countercurrents within the tumor microvessels. Tumor anemic hypoxia can be caused by the reduced oxygen transport capacity due to the tumor itself or chemotherapy-induced anemia. Generally, tumor hypoxia is independent of the size, stage, histopathological type, or grade of tumors.

Tumor cells can adapt to the hypoxic environment through activation of a large group of genes involved in different biological processes (Schito and Semenza, 2016; Petrova et al., 2018; Choueiri and Kaelin, 2020; de Heer et al., 2020; Lee et al., 2020). HIFs, a family of transcription factors composed of a heterodimer of an oxygen-dependent α-subunit and constitutively expressed β-subunit, play a key role in response to hypoxia. HIF-1α is the most well-studied member of the HIF family, which regulates the expression of genes involved in response to hypoxia in most mammalian cells (Schito and Semenza, 2016; Petrova et al., 2018). The other members of this family include HIF-2α, which also stabilizes under hypoxic conditions, and HIF-3α, which lacks the transactivation domain of HIF-1α and HIF-2α. Under the normoxic condition, HIF-1α is hydroxylated by the prolyl hydroxylases (PHDs) in its oxygen-dependent degradation domain (ODD) or by the factor inhibiting HIF (FIH) in its inhibitory domain, leading to the interaction of HIF-1α with the E3 ubiquitin ligase von Hippel-Lindau protein (pVHL) and ubiquitination and proteasomal degradation of HIF-1α (Huang et al., 2002; Paltoglou and Roberts, 2007; Schito and Semenza, 2016). The hypoxic condition inactivates PHDs, leading to HIF-1α stabilization and translocation to nuclear, where it forms a dimer with HIF-1β and binds to E-box-like hypoxia response elements (HREs) containing the consensus core sequence RCGTG (where R is A or G) (Kaelin and Ratcliffe, 2008; Schito and Semenza, 2016). HIF-2α is regulated in a similar manner to HIF-1α. HIF-1α and HIF-2α have a 48% amino acid sequence similarity, but they show different patterns of tissue distribution. While HIF-1α is ubiquitously expressed in different tissues, HIF-2α is highly expressed in vascular tissues such as the lung, heart, placenta, and kidney (Talks et al., 2000). The majority of HIF target genes are regulated by HIF-1α, whereas exclusively HIF-2α-dependent genes are scarce and cell type-dependent (Raval et al., 2005; Elvidge et al., 2006; Schito and Semenza, 2016). Interestingly, it was reported that HIF-1α is activated by acute and intense hypoxia, whereas HIF-2α is activated by chronic and mild hypoxia, suggesting that in some contexts HIF-1α plays a key role in the initial response to hypoxia, whereas HIF-2α drives the hypoxic response during chronic hypoxic exposure (Holmquist-Mengelbier et al., 2006; Koh et al., 2011; Kumar and Choi, 2015). The functions of HIF-3α and its splice variants are not clear; while some variants can activate gene expression, some variants act as negative regulators of HIF-1α and HIF-2α (Makino et al., 2002; Yang et al., 2015; Duan, 2016).

Through transcriptional regulation of HIF target genes, hypoxia regulates tumor progression in many different aspects, including cell survival and death, proliferation, angiogenesis, metastasis, metabolic reprogramming, stemness, immune evasion, and therapeutic resistance (Schito and Semenza, 2016; de Heer et al., 2020; Sun et al., 2020; Vito et al., 2020).

## p53 and Hypoxia

### Hypoxia Regulates p53

The regulation of p53 by the hypoxia and HIF signaling appears to be highly context-dependent. Many studies have reported that while HIF-1α accumulates under both moderate and severe hypoxia, p53 stabilization and activation usually occur under severe hypoxia. In many cells, it appears that while HIF-1α accumulation helps cells adapt to mild hypoxia, p53 accumulation and activation induce cell death under severe hypoxia. However, hypoxia and HIF have been reported to both activate and inactivate p53, which could be dependent on hypoxic conditions and types of cells and tissues as well.

Hypoxia has been reported to induce p53 protein levels and activities through both HIF-dependent and -independent manners. HIF-1α can regulate p53 through modulating MDM2 by several different mechanisms. It was reported that HIF-1α directly interacts with MDM2 but not p53 to block MDM2-mediated ubiquitination and nuclear export of p53 (Chen et al., 2003; Singh et al., 2017). As a HIF-1α target, PNUTS (protein phosphatase-1 nuclear targeting subunit) has the HRE in its promoter region and can be induced by hypoxia (Lee et al., 2007). The induction of PNUTS by hypoxia increases the ubiquitin-dependent proteasomal degradation of MDM2, leading to p53 activation and the p53-mediated apoptosis (Lee et al., 2007). Studies also reported that hypoxia decreases MDM2 levels partially through the activation of p38 MAPK *via* an unclear mechanism, enhancing p53 protein levels and transcriptional activities in neurons (Zhu et al., 2002). In addition to MDM2, hypoxia can induce p53 *via* regulation of other p53 negative regulators, such as E3 ubiquitin ligases CHIP and COP1. For instance, hypoxia represses the transcription of CHIP, leading to p53 accumulation in the heart after myocardial infarction (Naito et al., 2010). LncRNA Fendrr can bind to p53 protein and promote its interaction with COP1, leading to p53 ubiquitination and degradation (Li et al., 2020). Hypoxia decreases the levels of Fendrr, which in turn attenuates the p53–COP1 interaction and leads to the increase of p53 levels in myocardial cells (Li et al., 2020). Hypoxia also enhances p53 protein levels through activating ATR/ATM kinases independently of HIF-1α, leading to p53 phosphorylation at serine 15 and the resultant p53 stabilization and activation (Hammond et al., 2002, 2003). Furthermore, hypoxia stabilizes p53 through the production of mitochondrial reactive oxygen species (ROS) and hypoxia-associated acidosis (Schmaltz et al., 1998; Chandel et al., 2000; Humpton and Vousden, 2016). The RNA-binding protein HuR can bind to the p53 mRNA to promote its translation in response to ultraviolet light irradiation (Mazan-Mamczarz et al., 2003). HuR also increases HIF-1α levels through promoting the translation of HIF-1α mRNA (Galban et al., 2008; Wu et al., 2019). Interestingly, pVHL was reported to increase p53 protein levels through enhancing HuR levels and HuR-mediated p53 translation (Galban et al., 2003). However, it is unclear how pVHL upregulates HuR expression and whether pVHL-HuR-p53 signaling is involved in the hypoxia-triggered induction of p53 protein levels. In addition, hypoxia was reported to increase the mRNA levels of p53 in a HIF-1α-independent manner through an unclear mechanism (Wang et al., 2004).

**FIGURE 1:**
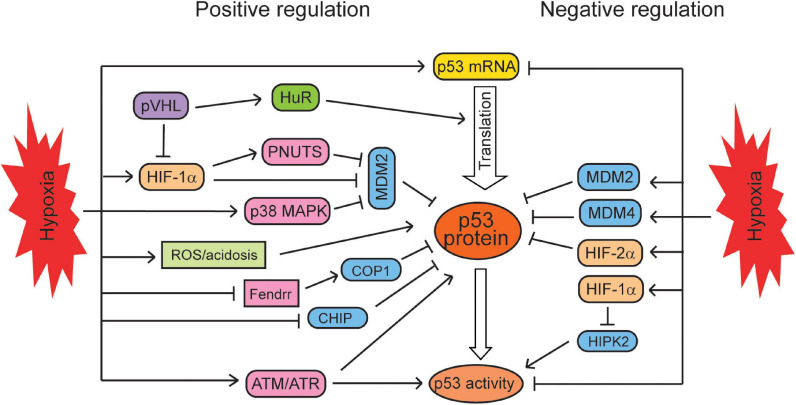
The regulation of the p53 signaling by hypoxia. Hypoxia regulates p53 in a context-dependent manner. Hypoxia positively regulates the mRNA levels, translation, protein levels, and activities of p53 through different mechanisms. At the same time, hypoxia can negatively regulate p53 mRNA levels, protein levels, and activities in certain types of cells under certain conditions.

The induction of p53 levels and transcriptional activities by hypoxia appears to be cell-type specific. Some studies reported that hypoxia and HIF signaling can negatively regulate p53 levels and activities in some cell lines. For instance, it was reported that in human cell lines treated with the chemotherapeutic agent etoposide, hypoxia induces p53 levels in breast cancer MCF7 cells, reduces p53 levels in liver cancer HepG2 cells, but does not affect p53 levels in lung cancer A549 cells under the same hypoxic condition (Cosse et al., 2007). HIF-1α was reported to directly repress the transcription of p53 in HeLa cells (Lee et al., 2001). Hypoxia also promotes the expression of two p53 negative regulators, MDM2 and MDM4, to downregulate p53 in human syncytiotrophoblasts and murine KHT fibrosarcoma cells (Zhang and Hill, 2004; Chen et al., 2010). Additionally, hypoxia was also reported to inhibit the phosphorylation of p53 at ser15 and ser392, reducing p53 activities in human syncytiotrophoblasts and normal human fibroblasts (Li et al., 2004; Chen et al., 2010). HIPK2 (serine/threonine homeodomain-interacting protein kinase 2) can phosphorylate p53 at ser46 to activate p53. In human prostate cancer cells and hepatocellular carcinoma cells, HIF-1α transcriptionally induces its targets to promote the proteasomal degradation of HIPK2, and thus, HIF-1α reduces p53 activities and p53-dependent apoptosis through downregulating HIPK2 (Nardinocchi et al., 2011; Chen et al., 2020). It was reported that p53 is stabilized only under the severe hypoxic condition (0.02% O_2_) but not under the mild hypoxic condition (2% O_2_) in human colorectal RKO cells, suggesting that the p53 activation by hypoxia is dependent on the severity of hypoxic conditions (Hammond et al., 2002; Humpton and Vousden, 2016). Taken together, hypoxia appears to regulate p53 in a cell/tissue type- and duration and severity of hypoxia-dependent manner, resulting in the increase or decrease of p53 levels and activities in cells (Figure 1).

### p53 Regulates the Hypoxia and HIF Pathway

**FIGURE 2:**
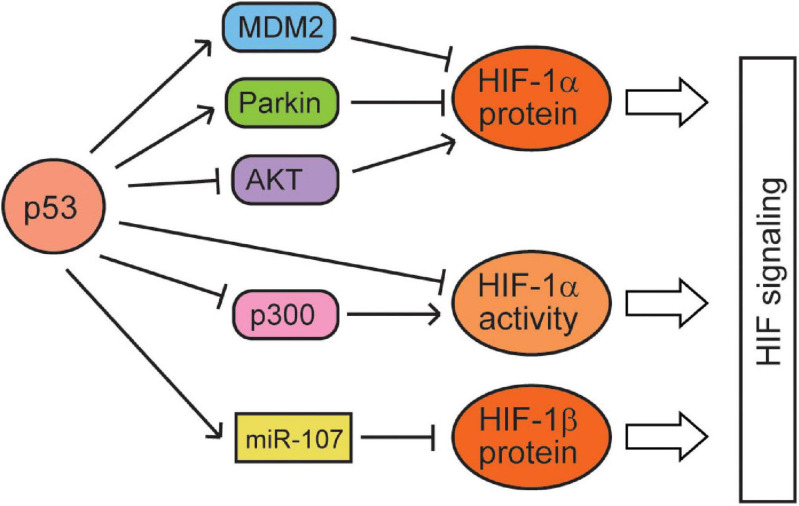
The regulation of the hypoxia and HIF signaling by p53. p53 represses the protein levels and activities of HIF-1α and HIF-1β through various mechanisms.

While hypoxia and the HIF signaling regulate p53 through many different mechanisms, p53 has also been reported to regulate the hypoxia and HIF signaling (Figure 2). p53 was reported to act as a scaffold protein to bridge MDM2 to HIF-1α, leading to the ubiquitination and proteasomal degradation of HIF-1α by MDM2 in HCT116 cells (Ravi et al., 2000; Singh et al., 2017). Blocking the interaction of p53 and MDM2 by the mutation of p53 in the transactivation domain (responsible for p53–MDM2 interaction) or treatment with nutlin-3 (a small-molecule MDM2 antagonist that disrupts MDM2–p53 interaction) leads to the accumulation of HIF-1α in p53 wild-type (WT) cells but not in p53-deficient cells, which supports that p53 negatively regulates HIF-1α through MDM2 (Ravi et al., 2000; Lee et al., 2009; Kojima et al., 2011). However, some studies also reported that MDM2 can increase the levels of HIF-1α (Nieminen et al., 2005; LaRusch et al., 2007), which suggests that the effect of MDM2 on HIF-1α may vary depending on the cell/tissue type and severity of hypoxia. Additionally, p53 can promote the ubiquitination and degradation of HIF-1α in an MDM2-independent manner in the transition from myocardial hypertrophy to cardiac dilatation and heart failure. However, its mechanism is unclear, and the inhibition of AKT phosphorylation may be involved in this process (Choy et al., 2010). p53 may also downregulate HIF-1α protein levels through another E3 ubiquitin ligase Parkin. Originally identified as a gene associated with neurodegenerative Parkinson’s disease, Parkin has been demonstrated to be a tumor suppressor (Liu et al., 2018). Parkin is a p53 target gene; p53 binds to the p53-binding element in the Parkin gene and transcriptionally induces Parkin expression (Zhang et al., 2011; Viotti et al., 2014). Interestingly, HIF-1α is an E3 ubiquitin ligase substrate of Parkin; Parkin binds to and ubiquitinates HIF-1α, leading to its proteasomal degradation (Liu et al., 2017b). Notably, treating Parkin-deficient cancer cells with small-molecule HIF-1α inhibitors greatly suppresses tumorigenesis of cancer cells in xenograft tumor models (Liu et al., 2017b). Furthermore, macrophage migration inhibitory factor (MIF), an inflammatory cytokine, can bind to p53 and sequester p53 from HIF-1α to block the MDM2-mediated degradation of HIF-1α, which in turn increases the HIF-1α protein levels under hypoxia (Oda et al., 2008). In addition to HIF-1α, p53 was also reported to reduce the levels of HIF-1β; p53 transcriptionally induces the expression of miRNA-107, which targets HIF-1β at its 3′-UTR to repress its expression, inhibiting the HIF-1 signaling in colorectal tumor cells (Yamakuchi et al., 2010).

In addition to regulating the HIF-1 protein levels, p53 also regulates the activities of HIF-1. Casein kinase 2 (CK2), a serine/threonine kinase, was reported to negatively regulate p53. It was reported that hypoxia activates CK2, which in turn increases the HIF-1 activities through reducing the p53 levels with an unclear mechanism (Hubert et al., 2006). It was reported that although high p53 expression reduces the HIF-1α protein levels, low p53 levels attenuate HIF-1 transcriptional activities by competing for p300, a coactivator required for the full activities of both p53 and HIF-1 (Blagosklonny et al., 1998; Schmid et al., 2004; Vleugel et al., 2006). Transfection of the full-length p300 stimulates transcriptional activities of both p53 and HIF-1, but does not relieve p53-mediated inhibition of HIF-1 transcription activities. In contrast, a p300 fragment that binds to p53 but not to HIF-1 prevents p53-dependent repression of HIF-1 activities (Blagosklonny et al., 1998; Ye et al., 2019). p53 with a mutation in its DNA binding domain (R273H) retains the ability to block the transactivation activity of HIF-1, whereas p53(22,23), a transcriptionally inactive double point mutant defective for p300 binding, does not inhibit HIF-1 (Blagosklonny et al., 1998). Furthermore, HIF-1α induces expression of Cockayne syndrome B (CSB), which competes with p53 for p300, leading to the redistribution of p300 between p53 and HIF-1 (Filippi et al., 2008; Ye et al., 2019). Under mild hypoxia, HIF-1α dissociates p300 from p53 to promote HIF-1 activation through inducing CSB, whereas under severe hypoxia, the accumulation of p53 in cells wins the competition for p300, leading to the inhibition of HIF-1 activities. Thus, while HIF-1α accumulation induces its targets, leading to the adaptation of cells to mild hypoxia, p53 accumulation and activation induce cell death under severe hypoxia (Figure 2).

## Biological Outcomes of the Interplay Between p53 and Hypoxia Pathways

**FIGURE 3:**
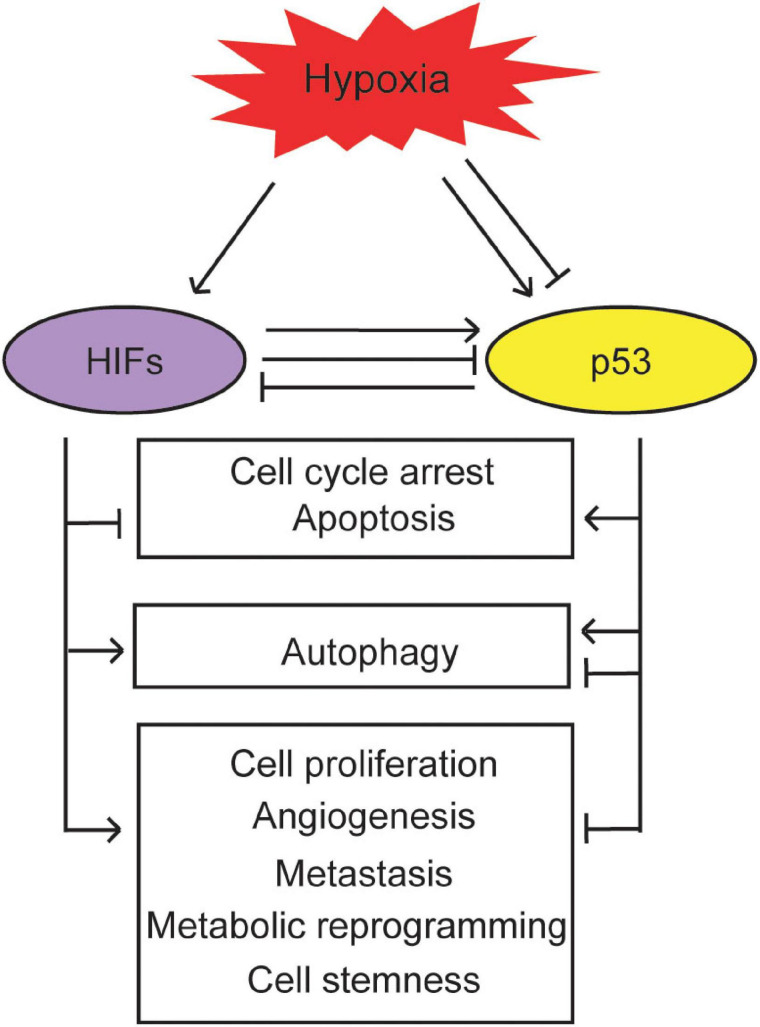
The biological outcomes of the interplay between the p53 and hypoxia signaling pathways. p53 cross-talks with hypoxia to regulate cell cycle arrest, apoptosis, autophagy, cell proliferation, angiogenesis, metastasis, metabolic reprogramming, and cell stemness in cancer.

Activation of the HIF pathway by hypoxia contributes to tumor progression through many biological processes, including cell survival and proliferation, angiogenesis, metastasis, metabolic reprogramming, and cell stemness. As summarized above, the p53 pathway cross-talks with the hypoxia and HIF pathway. This cross-talk impacts biological outcomes in response to hypoxia in tumors (Figure 3).

### Cell Survival and Proliferation

Tumor progression is associated with enhanced cell proliferation and decreased cell death. Many studies have suggested that HIF-1 inhibits cell death, including apoptosis and autophagy, and promotes cell proliferation (Kumar and Choi, 2015). In contrast, p53 activation often leads to cell cycle arrest and apoptosis (Levine et al., 2006; Vousden and Prives, 2009; Levine, 2019). Studies have reported a controversial role of hypoxia in apoptosis, which appears to be dependent on the oxygen concentration: oxygen levels in the range 0–0.5% in cells induce apoptosis, while oxygen levels in the range of 1–3% in cells do not induce apoptosis (Santore et al., 2002; Kumar and Choi, 2015). It has been reported that p53 is usually activated under severe hypoxia or anoxia, leading to rapid apoptosis; whereas under mild hypoxia, p53 is constrained and tends to support cell survival (Li et al., 2004; Hammond and Giaccia, 2005; Feng et al., 2011; Humpton and Vousden, 2016). As an important regulator of apoptosis, p53 induces its target Bax, which in turn initiates the cascade, leading to cytochrome c release and apoptosis (Wei et al., 2001; Levine, 2019). Cells with deficiency of pro-apoptotic proteins Bax and Bak are resistant to hypoxia-induced apoptosis, whereas overexpression of anti-apoptotic proteins Bcl-2 or Bcl-XL prevents hypoxia-induced apoptosis by inhibiting the cytochrome c release (McClintock et al., 2002; Moldoveanu and Czabotar, 2020). Severe hypoxia induces the expression of pro-apoptotic gene BNIP3 *via* HIF-1α, resulting in hypoxia-induced apoptosis (Guo et al., 2001; Gorbunova et al., 2020). Under mild hypoxia, p53 directly suppresses BNIP3 expression to protect cells against hypoxia-induced apoptosis (Feng et al., 2011). Under severe hypoxia, p53 induces apoptosis through inducing the expression of another pro-apoptotic gene BNIP3L, a homolog of BNIP3, which contributes to the role of p53 in tumor suppression (Fei et al., 2004; Gorbunova et al., 2020).

In addition to apoptosis, hypoxia induces autophagy *via* both HIF-1-dependent and -independent pathways, such as the mTOR pathway, unfolded protein response (UPR) pathway and PKCδ-JNK1 pathway (Fang et al., 2015; Daskalaki et al., 2018). Autophagy plays a protective role by mediating the removal of the damaged organelles and proteins under chronic and mild hypoxia, whereas it may be detrimental and induce cell death during rapid and severe hypoxia (Fang et al., 2015). p53 was reported to facilitate autophagy by inducing the expression of DRAM, Sestrins, and AMPK and inhibiting the mTOR pathway (Crighton et al., 2006; Feng and Levine, 2010; Xu-Monette and Young, 2012). BNIP3 and BNIP3L, which are induced under hypoxia *via* HIF-1α (Gorbunova et al., 2020), also play important roles in promoting autophagy in addition to apoptosis (Bellot et al., 2009; Daskalaki et al., 2018). Ectopic expression of BNIP3 and BNIP3L can activate autophagy by disrupting the Bcl-2–Beclin1 complex (Bellot et al., 2009; Gorbunova et al., 2020). p53 induces BNIP3 expression and leads to autophagy (Wang et al., 2013). Furthermore, p53 also induces expression of BNIP3L under hypoxia, which promotes autophagy (Fei et al., 2004; Bellot et al., 2009; Gorbunova et al., 2020). However, the role of p53 in autophagy appears to be context dependent. It was also reported that the cytoplasmic fraction of p53 represses autophagy (Tasdemir et al., 2008). Studies have suggested that autophagy has dual functions in both tumor suppression and promotion (White, 2015; Yun and Lee, 2018). Therefore, it will be important to understand the precise role of autophagy under different hypoxic conditions in different cells and tissues.

Hypoxia also regulates cell proliferation in addition to cell death. VEGF is an important target gene of the HIF pathway involved in cell proliferation, which is upregulated in many types of cancers (Barak et al., 2011; Schito and Semenza, 2016; Choueiri and Kaelin, 2020; Lee et al., 2020). HIF-2α was reported to promote the cell cycle progression through regulation of its target Cyclin D1 and indirect regulation of p21 and p27, and by enhancing c-Myc function as well (Barak et al., 2011; Schito and Semenza, 2016; Choueiri and Kaelin, 2020; Lee et al., 2020). In contrast, p53 was reported to induce cell cycle arrest of hypoxic cells through transcriptional activation of p21 (Yu et al., 2003; Levine, 2019).

### Angiogenesis

Angiogenesis is one of the classic responses to hypoxia. HIF-1α can induce the expression of several pro-angiogenic factors, including VEGF and PDGF (platelet-derived growth factor), to promote angiogenesis (Schito and Semenza, 2016; Choueiri and Kaelin, 2020; de Heer et al., 2020). As a tumor suppressor, p53 is known to inhibit angiogenesis, and p53 loss or inactivation promotes hypoxia-induced angiogenesis in both HIF-dependent and -independent manners (Ravi et al., 2000; Levine, 2019). Consistent with the regulation of p53 by hypoxia, the regulation of angiogenesis by p53 under the hypoxic condition also appears to be context dependent. While p53 can activate angiogenesis in the initial hypoxic phase, p53 suppresses angiogenesis as a tumor-suppressive mechanism under persistent hypoxic conditions (Farhang Ghahremani et al., 2013). Loss of p53 in tumor cells leads to enhanced HIF-1α levels and HIF-1-dependent VEGF activation upon hypoxia, which in turn augments neovascularization and tumor growth (Ravi et al., 2000; Schito and Semenza, 2016; de Heer et al., 2020). p53 also decreases the levels of HIF-1β by transcriptionally inducing miRNA-107, which in turn reduces VEGF expression, leading to the inhibition of angiogenesis (Yamakuchi et al., 2010). Interestingly, it was also reported that under acute hypoxia, p53 can bind to the p53-binding element in the promoter region of VEGF in a HIF-1α-dependent manner, enhancing VEGF expression and angiogenesis, whereas under chronic hypoxia, the expression of VEGF can be repressed by the retinoblastoma (Rb) pathway in a p21-dependent manner (Farhang Ghahremani et al., 2013). On the contrary, HIF-1α reduces HIPK2 levels to inhibit p53 activities, which in turn enhances angiogenesis in hepatocellular carcinoma (Chen et al., 2020).

### Metastasis

Hypoxia also plays an important role in promoting metastasis, which is the primary reason for cancer-related mortality. Many target genes of HIFs are related to cancer metastasis, and HIFs were reported to regulate different aspects of metastasis (Keith et al., 2011; Balamurugan, 2016; de Heer et al., 2020). p53 plays a key role in suppression of cancer metastasis (Vousden and Prives, 2009; Muller and Vousden, 2014; Levine, 2019; Zhang et al., 2020). Parkin, as a p53 target, inhibits metastasis of breast cancers through direct binding to HIF-1α to lead to HIF-1α ubiquitination and degradation (Liu et al., 2017b). p53 was also reported to decrease migration, invasion, and metastasis of cancer cells *via* another p53 target GLS2 (Zhang et al., 2016). GLS2 is a mitochondria glutaminase, which converts glutamine to glutamate. GLS2 expression can be induced by p53 activation in response to ROS, which is often a consequence of hypoxia (Suzuki et al., 2010). GLS2 mediates the role of p53 in regulating mitochondrial function and antioxidant defense in cells (Hu et al., 2010; Suzuki et al., 2010). Interestingly, GLS2 directly binds to small GTPase Rac1 independent of its glutaminase activities, which in turn inhibits Rac1 activation to suppress cancer metastasis (Zhang et al., 2016).

### Metabolic Reprogramming

Metabolic reprogramming is a hallmark of cancer, which plays a key role in cancer progression. The most well-known metabolic change in cancer is the Warburg effect, which is characterized by the enhanced glycolysis under the normoxic condition and is crucial for cancer progression (Liu J. et al., 2019; Hoxhaj and Manning, 2020). Hypoxia induces similar metabolic changes as the Warburg effect. HIF-1 activation promotes glycolysis by enhancing the expression of genes encoding glucose transporters (GLUTs) and glycolytic enzymes such as hexokinase 1/2, phosphoglycerate mutase 1, enolase 1, and pyruvate kinase M2 (Gonzalez et al., 2018; Kierans and Taylor, 2020). HIF-1 can also inactivate the pyruvate dehydrogenase complex *via* promoting the expression of pyruvate dehydrogenase kinases (PDKs), which in turn decreases the flux of pyruvate into the tricarboxylic acid (TCA) cycle and mitochondrial respiration, and increases its flux to the lactate (Kim et al., 2006; Papandreou et al., 2006). p53 has been reported to play an important role in inhibiting glycolysis through transcriptional regulation of its targets, such as TIGAR, Parkin, hexokinase 2, phosphoglycerate mutase 1, and RRAD (Zhang et al., 2011, 2014; Kruiswijk et al., 2015; Liu J. et al., 2019). Under the hypoxic condition, p53 activation leads to the induction of the expression of its target RRAD, a small GTPase. RRAD binds to p65 and inhibits the NF-kB signaling, which suppresses GLUT1 translocation to the cell surface to reduce glucose uptake in cells (Zhang et al., 2014).

In addition to glycolysis, hypoxia has also been reported to impact other metabolic pathways. Hypoxia induces the glycosylation and activation of glucose-6-phosphate dehydrogenase (G6PD), the rate-limiting enzyme of the pentose phosphate pathway (PPP), to promote the PPP and tumor growth (Rao et al., 2015). Interestingly, p53 inhibits G6PD activities by preventing the formation of the active G6PD dimer (Jiang et al., 2011). Hypoxia also modulates lipid metabolism. Hypoxia can promote acetate-dependent epigenetic activation of lipogenic genes such as acetyl-CoA carboxylase α (ACACA) and fatty acid synthase (FASN) to enhance the uptake of fatty acids and *de novo* lipid synthesis (Gao et al., 2016). Hypoxia elevates glutamine-dependent lipid synthesis *via* the degradation of α-ketoglutarate dehydrogenase (αKGDH) and carboxylation of α-ketoglutarate (α-KG) (Wise et al., 2011; Sun and Denko, 2014). p53 transcriptionally inhibits SREBP1c, a transcription factor that activates the transcription of many lipogenic genes (Yahagi et al., 2003). Further, p53 transcriptionally induces the expression of ABCA1, a transporter controlling retrograde cholesterol transport, leading to the inhibition of maturation and nuclear translocation of SREBP2, another transcription factor that mainly contributes to the lipid synthesis *via* the mevalonate pathway (Moon et al., 2019).

Additionally, HIF1α can suppress fatty acid β-oxidation by regulating the expression of medium/long-chain acetyl-CoA dehydrogenases (M/LCADs) and fatty acid binding proteins (FABPs) (Bensaad et al., 2014; Huang et al., 2014). On the contrary, p53 promotes fatty acid β-oxidation by inducing the expression of carnitine *O*-octanoyltransferase (CROT) that facilitates fatty acids efflux out of the peroxisome (Goldstein et al., 2012), and the expression of the transporters that convey fatty acids into the mitochondria (CPT1A and CPT1C) (Sanchez-Macedo et al., 2013), as well as the expression of several genes that are directly or indirectly involved in the fatty acid β-oxidation (e.g., ACAD11, HMGCLL1, and MCD) (Liu et al., 2014; Jiang et al., 2015). Further, hypoxia increases both ROS and HIF activities as a function of decreasing oxygen levels (Chandel et al., 1998; Bell et al., 2007). In response to ROS, p53 promotes antioxidant defense through inducing the expression of a group of antioxidant genes, including Sestrins 1/2, TIGAR, GPX1, ALDH4, GLS2, and Parkin (Berkers et al., 2013; Liang et al., 2013). For instance, p53 enhances the intracellular levels of glutathione (GSH) to reduce ROS levels by inducing its target GLS2 and Parkin (Hu et al., 2010; Suzuki et al., 2010; Zhang et al., 2011). Further, p53 reduces ROS levels in cells by promoting the stabilization of NRF2, a transcription factor that plays a critical role in antioxidant defense, through its upregulation of p21 (Chen et al., 2009).

### Cell Stemness

A small part of the heterogeneous cancer cell population with high self-renewal potential, which is known as cancer stem cells (CSCs), is responsible for tumor initiation, recurrence, and metastasis. CSCs are resided in a specific tissue niche, forming complicated interactions with the supporting cells and microenvironment factors, such as hypoxia. Hypoxia can promote the stem cell-like phenotype in cancer cells and increase the expansion of CSCs in cancers (Soeda et al., 2009; Bar et al., 2010; Sun et al., 2020). It was reported that HIF-1α activation promotes the expansion of CD133-positive CSCs in brain and pancreatic cancers, whereas HIF-1α deletion decreases leukemia stem cell capacity (Soeda et al., 2009; Bar et al., 2010; Hashimoto et al., 2011; Sun et al., 2020). Similarly, HIF-2 was reported to induce the expression of genes related to the stem cell function, such as Oct-4 in cancer cells under hypoxia (Covello et al., 2006; Sun et al., 2020). p53 also regulates the stemness of both normal and cancer cells. p53 can inhibit the reprogramming of differentiated somatic cells to induced pluripotent stem cells (iPSCs) by inducing the expression of p21 and miRNAs (e.g. miR-34), and repressing the expression of stem cell markers, such as Oct-4, Sox-2, and Nanog (Levine et al., 2016; Koifman et al., 2019). Compared with normoxia, hypoxia is favorable for maintaining the stemness of human endothelial progenitor cells, which shows the low activities of the p53 signaling (Lin et al., 2019). In human embryonic stem cells, HIF-2α was reported to suppress p53 to promote the expression of Nanog, leading to the reprogramming of SSEA3+ /ABCG2+ cells to an even higher state of stemness (Das et al., 2012). Furthermore, physiological hypoxia (10% O_2_) enhances the stemness properties and promotes the proliferation ability of induced hepatic stem cells by inhibiting the p53–p21 signaling pathway to accelerating G1/S transition (Zhi et al., 2018).

In sum, the hypoxia/HIF and p53 pathways can cross-talk at different levels. This cross-talk appears to be highly cell type- and context-dependent. Furthermore, as a tumor suppressor, p53 antagonizes the oncogenic effects of hypoxia in different aspects.

## Gof Mutant p53 and Hypoxia

Most of p53 mutations in cancers are missense mutations, which encodes the full-length mutant p53 proteins. In addition to the loss of WT p53 function as a tumor suppressor, many tumor-associated mutant p53 proteins display oncogenic activities, which is known as gain-of-function (GOF), to promote tumor progression by regulating cell proliferation, survival, metastasis, metabolic reprogramming, genomic instability, stemness, tumor microenvironment adaption, and immune evasion (Muller and Vousden, 2014; Donehower et al., 2019; Levine, 2019; Zhang et al., 2020). These mutant p53 proteins often accumulate to very high levels in cancer cells through different mechanisms, including posttranslational modifications (such as ubiquitination, acetylation, and phosphorylation), interaction with chaperones and co-chaperone proteins, as well as induction by different stress signals (Muller and Vousden, 2014; Yue et al., 2017; Donehower et al., 2019; Zhang et al., 2020). Hypoxia has been reported to increase GOF mutant p53 protein levels (Mantovani et al., 2019; Zhang et al., 2020). Although it is still unclear how hypoxia enhances mutant p53 levels, some mechanisms by which hypoxia induces WT p53 may also contribute to the induction of mutant p53 in cells (Yamamoto and Iwakuma, 2018). It was reported that tumors bearing p53 mutations are generally characterized by higher HIF-1α levels, and mutant p53 appears to stimulate HIF-1α stabilization by blocking its interaction with MDM2 under the hypoxic condition (Kamat et al., 2007).

Gain-of-function mutant p53 can promote the adaptation of cancer cells to hypoxia. Mutant p53 activates several intracellular signaling pathways to promote angiogenesis, which helps the survival of cancer cells under the hypoxic condition. It was reported that mutant p53 activates protein kinase C (PKC) to increase the expression of VEGF, which in turn promotes angiogenesis (Kieser et al., 1994; Zhang et al., 2020). Mutant p53 induces VEGF expression through forming a complex with the lncRNA MALAT1 to promote the chromatin association of MALAT1, leading to recruitment of MALAT1 on VEGFA pre-mRNA to enhance its pro-angiogenic isoform expression in breast cancer cells (Pruszko et al., 2017). Mutant p53 forms a complex with E2F1 and induces ID4 (inhibitor of DNA-binding 4) to enhance the expression of pro-angiogenic factors IL8 and GRO-α, which promotes angiogenesis (Fontemaggi et al., 2009). Mutant p53 also promotes tumorigenesis through the HIF pathway. It was reported that loss of one allele of HIF-1α, but not HIF-2α, in a mutant p53 mouse model (p53 R270H/R270H) reduces the incidence of thymic lymphomas (Bertout et al., 2009). Further, mutant p53 forms a complex with HIF-1α, which in turn binds to the SWI/SNF chromatin-remodeling complex and induces the expression of a selective subset of its target genes, including VEGFR2 (VEGF receptor 2), to promote angiogenesis (Pfister et al., 2015). The mutant p53-HIF-1α complex also induces expression of some extracellular matrix (ECM) genes such as type VIIa1 collagen (COL7A1) and laminin-γ2 (LAMC2) (Amelio et al., 2018). Depletion of mutant p53 impairs hypoxia-mediated metastasis of non-small cell lung cancer (NSCLC) cells, which can be reverted by overexpression of COL7A1 and LAMC2. Consistently, the higher levels of COL7A1 and LAMC2 are correlated with HIF-1 activation in NSCLC carrying p53 mutations, and associated with a poor prognosis of the patients (Amelio et al., 2018). Mutant p53 was reported to promote metastasis of cancer cells by regulating different pathways, including ZEB1, Rac1, EGFR, NF-Y, and Smad2/3 pathways, which may allow tumor cells to escape from the hypoxic environment (Pitolli et al., 2019; Zhang et al., 2020). In addition, mutant p53 interacts with p63 to inhibit the expression of Sharp1, an anti-metastatic p63 target gene. Sharp1 promotes ubiquitination-mediated degradation of HIF-1α and HIF-2α to attenuate HIF-induced malignant cell behavior (Adorno et al., 2009; Montagner et al., 2012; Zhang et al., 2020).

## p53 and Hypoxia in Cancer Therapy

Since over 50% of human tumors contain p53 mutations including many GOF mutations, and over 80% of human tumors are estimated to have the impaired p53 function, p53 has become a very attractive target for cancer therapy. Many strategies have been tested and developed to restore WT p53 function and/or block GOF mutant p53 function and signaling in tumors (Sabapathy and Lane, 2018; Zhou et al., 2019; Zhang et al., 2020). As a hallmark of the majority of solid tumors, hypoxia is associated with insufficient response to standard cancer therapies and poor prognosis of cancer patients (Baran and Konopleva, 2017; Albadari et al., 2019; Spiess, 2020). Therefore, like p53, the hypoxia/HIF signaling pathway is an attractive target for cancer therapy. Currently, several hypoxia-activated prodrugs that selectively kill tumor cells in hypoxic zones of tumors, and oxygen-carrier compounds that reverse the effects of aberrant tumor vasculature, as well as small-molecule inhibitors of both HIF-1 and HIF-2 are available (Baran and Konopleva, 2017; Albadari et al., 2019; Spiess, 2020; Schonberger et al., 2021). Given that loss of p53 function and hypoxia are common events in solid tumors, targeting p53 and hypoxia simultaneously has been tested as a promising strategy for cancer therapy. Based on the finding that HIF-1α interacts with p53 and inhibits its transcriptional activity in hypoxic cancer cells, Topotecan (TPT), a topoisomerase inhibitor used in ovarian cancer treatment, was reported to downregulate HIF-1α in hypoxic cells to enhance p53 transcriptional activities and restore p53 tumor-suppressive function, which may offer a novel approach to reverse hypoxia-related cisplatin and paclitaxel resistance in ovarian cancers expressing WT p53 (Parmakhtiar et al., 2019). RITA, a small-molecule that activates WT p53 through blocking MDM2-p53 interaction, can induce p53 and inhibit expression of HIF-1α and VEGF *in vivo* and induce apoptosis of tumor cells under hypoxia in tumor cells expressing WT p53 (Yang et al., 2009). TAT-ODD-p53, a p53 fusion protein conjugated with the minimum motif of oxygen-dependent degradation domain (ODD) and the basic domain of the TAT (transactivator of transcription) protein of HIV-1, was reported to induce the cell cycle arrest and apoptosis to inhibit the growth of human lung H1299 cancer cells in a p53-dependent manner especially under hypoxia *in vitro*. Furthermore, TAT-ODD-p53 selectively accumulates in the hypoxic areas of solid tumor tissues and inhibits the growth of xenograft tumors in a p53-dependent manner (Zhao et al., 2011). This strategy may be particularly useful for hypoxic tumors with WT p53 deficiency. Recently, a manganese-clay hybrid compound (MHC), which targets hypoxia by generating molecular oxygen in aqueous solution, was shown to induce p53-dependent apoptosis under hypoxia, suggesting that MHC can be used to treat hypoxic tumors containing WT p53 (Deepa et al., 2020). p53 contains a single zinc ion near its DNA-binding interface, which is critical for p53 conformation and transcriptional activity. Supplementation of zinc was reported to restore the WT DNA-binding activities of mutant p53 to reactivate the p53-induced apoptosis in response to chemotherapeutic drugs (Puca et al., 2011). Zinc supplementation was shown to increase the stability and nuclear translocation of HIPK2, which binds to the HIF-1α promoter to repress transcriptional activities of HIF-1 (Nardinocchi et al., 2009). Based on these findings, targeting both hypoxia and mutant p53 by zinc supplementation was used in combination with other chemotherapeutic drugs to improve the treatment for hypoxic tumors expressing mutant p53 (Nardinocchi et al., 2009). Inhibition of the proteins regulating the G2/M checkpoint, such as Chk1, has been shown to potentially induce synthetic lethality in tumors expressing mutant p53 (Zhou et al., 2019; Zhang et al., 2020). Interestingly, inhibition of Chk1 was shown to enhance the anti-tumor activity of hypoxia-activated prodrug TH-302, suggesting that the combination of CHK1 inhibitors and TH-302 could be a potential treatment for hypoxic tumors expressing mutant p53 (Meng et al., 2015). These studies provide evidence of targeting the hypoxia/HIF and p53 signaling pathways in cancer simultaneously for improved cancer treatment, which will inspire many future studies and better strategies in cancer therapies.

## Discussion

Both hypoxia and p53 signaling pathways in cancer have been extensively studied for decades. Although many studies have demonstrated the close interplay between hypoxia and p53 signaling pathways, our current understanding of this interplay and its impact on cellular responses, tumor progression, and cancer therapy is still far from clear. Hypoxia and the HIF pathway play important roles in tumor progression through promoting cell proliferation, angiogenesis, metastasis, metabolic reprogramming, and cell stemness. As a tumor suppressor, p53 often negatively regulates these cellular processes to display its tumor suppressive function. However, cellular responses to hypoxia appear to be extremely complicated, depending on the duration and severity of the hypoxia as well as cell and tissue types. It appears that in some types of cells and tissues, severe hypoxia increases p53 levels and activities to induce cell death, whereas mild hypoxia decreases p53 levels and activities to promote cell survival. Although many mechanisms have been proposed on how hypoxia regulates p53, it is still not clear how this context-dependent regulation is achieved, which may involve the selective transactivation of different p53 target genes under different conditions. Similarly, although some recent studies have revealed that mutant p53 coordinates with the hypoxia and HIF pathway and helps the cancer cells to adapt to the hypoxic environment, which is consistent with the GOF oncogenic effect of mutant p53, our understanding of the interplay between mutant p53 and the hypoxia and HIF pathway is still very limited. Additionally, it is also worth noting that the physiological hypoxia and the conditions used in the *in vitro* culture experiments to mimic physiological hypoxia *in vivo* are not the same. Many studies used anoxic conditions (nearly 0% oxygen) or chemicals to stabilize HIF, such as cobalt chloride or deferoxamine, to mimic the hypoxic conditions in their experiments, which could be different from the hypoxic conditions in tumors and other diseases. Using the non-invasive electron paramagnetic resonance imaging technique, it was reported that the oxygen concentration in solid tumors varies from 0.3 to 2.2% (Bratasz et al., 2007; Muz et al., 2015). Furthermore, many of these studies on the interplay between p53 and hypoxia were performed in *in vitro* cell culture systems using different cell lines, including many cancer cell lines containing mutations of different genes. Different genetic or epigenetic backgrounds of these cancer cell lines may affect the interplay between p53 and hypoxia/HIF signaling pathways. Future studies should better mimic hypoxic conditions in tumors and use *in vivo* animal models to further elucidate the interplay between hypoxia and p53 in tumors and its underlying mechanisms. Given that the loss of p53 tumor-suppressive function and hypoxia are two common biological events observed in solid tumors, targeting p53 and hypoxia simultaneously could be a promising strategy for cancer therapy, which is being actively tested. In addition to cancer, both hypoxia and p53 have been shown to play important roles in other physiological and pathological processes, including immunity, inflammation, tissue ischemia/reperfusion injuries, reproductive defects, neurodegenerative diseases, and aging. Therefore, a better understanding of the interplay between hypoxia and p53 pathways, the underlying molecular mechanisms, and its impact on these afore-mentioned biological processes and diseases will lead to novel and effective therapeutic strategies for cancer and other diseases.

## Author Contributions

CZ, JL, JW, TZ, and DX wrote the original draft. WH and ZF edited and reviewed the final draft. All authors read and approved the published version of the manuscript.

## Conflict of Interest

The authors declare that the research was conducted in the absence of any commercial or financial relationships that could be construed as a potential conflict of interest.
